# Orthopedic Biomaterials in the Clinical Reconstruction of Osteoporotic Atlantoaxial Injuries: A Review

**DOI:** 10.3390/bioengineering13080901

**Published:** 2026-08-10

**Authors:** Xiaohu Zhang, Jie Liu, Zilin Gao, Xiaohui Wang

**Affiliations:** 1 Honghui Hospital, Xi’an Jiaotong University, Xi’an 710054, China; erroneous1998@163.com (X.Z.); 17609259098@163.com (J.L.); 2 Graduate School, Xi’an Medical University, Xi’an 710021, China

**Keywords:** bioengineering, osteoporosis, atlantoaxial spine, spinal implant screw, low-modulus titanium alloy, bioactive coating, bone–implant interface augmentation

## Abstract

Background: Aging of the population increases the incidence of osteoporosis with atlantoaxial instability. Poor bone microarchitecture causes high rates of screw loosening, implant failure, and revision surgery, which remains a critical challenge for upper cervical surgery. Traditional atlantoaxial fixation is less effective in those with low bone mineral density, while thoracolumbar screw augmentation techniques are limited by the complex craniocervical anatomy. Methods: This review systematically summarizes the mainstream screw optimization strategies from a bioengineering perspective, including low-modulus alloy substrate modification, bionic threaded structural design, bioactive surface coatings, expandable screws, and cement-augmented cannulated screws. We compare the biomechanical anchorage performance, osteogenic capacity, and clinical complications of each technique. Results: Low-elastic-modulus titanium alloys reduce stress shielding; gradient multi-thread, bicortical, and CBT trajectory designs significantly improve anti-pullout stability; HA, tantalum, and composite extracellular matrix coatings promote osseointegration; and expandable screws and fenestrated cement screws can achieve reliable reinforcement. However, a narrow atlantoaxial space restricts the application of cement augmentation and expandable implants, and long-term, large-sample clinical evidence for the efficacy of novel screws is insufficient. Conclusion: Patient-specific porous degradable screws, which combine 3D-printed biomaterials, gradient alloys, and sustained-release bioactive coatings, represent a promising future research direction. This review provides theoretical support for the development of atlantoaxial internal fixation devices targeting elderly osteoporotic patients.

## 1. Introduction

The atlantoaxial segment (C1–C2) serves as the core functional unit responsible for cranial rotation within the craniocervical junction. The distinctive ring-shaped atlas, the odontoid process of the axis, and the ligamentous complex collectively maintain craniocervical stability [1]. Trauma, degenerative changes, congenital malformations, and inflammatory lesions can disrupt the osseous and ligamentous restraining structures, thereby resulting in atlantoaxial subluxation and instability. In cases with severe displacement, the odontoid process may compress the upper cervical spinal cord, leading to limb weakness, sensory deficits, or even life-threatening neurological impairment [2,3]. Posterior screw fixation has become the predominant surgical procedure to restore local mechanical equilibrium and protect the spinal cord. In this qualitative narrative review, we comprehensively retrieved peer-reviewed original biomechanical studies, animal experiments, and clinical cohort studies focusing on atlantoaxial fixation and spinal implant modification. Instead of quantitative meta-analysis, we integrated, compared, and interpreted the existing data from a bioengineering–material perspective, providing a targeted reference for upper cervical spine surgeons and biomaterial researchers.

With progressive population aging, the number of elderly patients diagnosed with atlantoaxial disorders and osteoporosis has continued to increase. As a systemic metabolic bone disease, osteoporosis is characterized by bone mass loss, sparse and fractured trabeculae, thinned cortical bone, and reduced osteogenic activity [4,5]. These pathological alterations directly impair the anchorage interface between screws and host bone and drastically reduce the screws’ pullout resistance and anti-fatigue performance [6,7]. Clinical evidence indicates that the incidence of screw loosening in atlantoaxial fixation among osteoporotic patients can reach up to 60%. Such complications often lead to implant failure and recurrent deformity, which frequently necessitate revision surgery and substantially increase surgical morbidity and clinical risks [8,9].

At present, most existing reviews on atlantoaxial internal fixation at home and abroad have focused on clinical surgery, mainly elaborating on key anatomical points and operative techniques for various screw placement methods [10,11,12]. Few systematic reviews have summarized implant modification strategies from a bioengineering perspective. Mature screw reinforcement techniques, including thread redesign, novel alloy substrates, bioactive coatings, and cement augmentation, have been widely studied for the thoracolumbar spine. However, the atlantoaxial region features a narrow anatomical space adjacent to vital structures such as the vertebral artery, spinal cord, and nerve roots, resulting in an extremely low surgical error tolerance for this region. Direct transplantation of thoracolumbar augmentation techniques may cause severe complications such as cement leakage and neurovascular injury. Despite this, there are no comprehensive overviews of implant optimization systems tailored for osteoporotic upper cervical spines.

This review systematically summarizes, from a bioengineering perspective, a diversity of modification approaches, including screw structural design, metallic substrate improvement, surface functional coating, and screw tract augmentation. We compare the biomechanical behavior and osteogenic outcomes of different strategies. Considering the advances in cutting-edge biomaterials, we also prospect the development trend of next-generation screws. This work provides theoretical support for the optimized design of atlantoaxial implants used in patients with osteoporosis. Distinct from previous clinical technique-focused atlantoaxial reviews, this review uniquely interprets implant anchorage failure in osteoporosis patients via bioengineering principles. Readers, including spinal surgeons, orthopedic biomaterial engineers, and spine researchers, can quickly understand the pros, cons, and upper cervical application restrictions of all mainstream modified screw systems, as well as the unresolved research bottlenecks worthy of further exploration.

## 2. Overview of Conventional Posterior Atlantoaxial Internal Fixation Systems

Three classic configurations dominate conventional posterior atlantoaxial internal fixation in clinical practice: Magerl transarticular screws, screw–plate constructs, and the Harms screw–rod system. All these techniques rely on screws to achieve rigid segmental fixation, yet their anchoring performance is markedly worse in osteoporotic bone [13,14,15]. Bilateral C1–C2 Magerl transarticular screws possess excellent anti-rotation and anti-lateral bending biomechanical properties. Their fusion rate exceeds 95% with bone grafting alone and approaches 100% when combined with cable fixation, which has made this technique the long-standing “gold standard” for atlantoaxial stabilization [10,16]. However, anatomical variations in the vertebral artery preclude bilateral screw placement in approximately 20% of patients due to the risk of vertebral artery or nerve root injury. Severe osteoporosis and irreducible atlantoaxial deformity are absolute contraindications for this procedure [14,17].

Representative screw–plate systems include the Goel lateral mass plate, modified occipitocervical plates, and posterior C1 locking plates combined with C2 laminar screws. These implants allow for intraoperative reduction of the atlantoaxial joint with satisfactory fixation stiffness. Nevertheless, the classic Goel technique requires transection of the C2 ganglion, which leads to a high incidence of postoperative scalp numbness. New locking plates have only undergone in vitro biomechanical testing, and relevant clinical data are not yet available [11,18,19].

**Figure 1:**
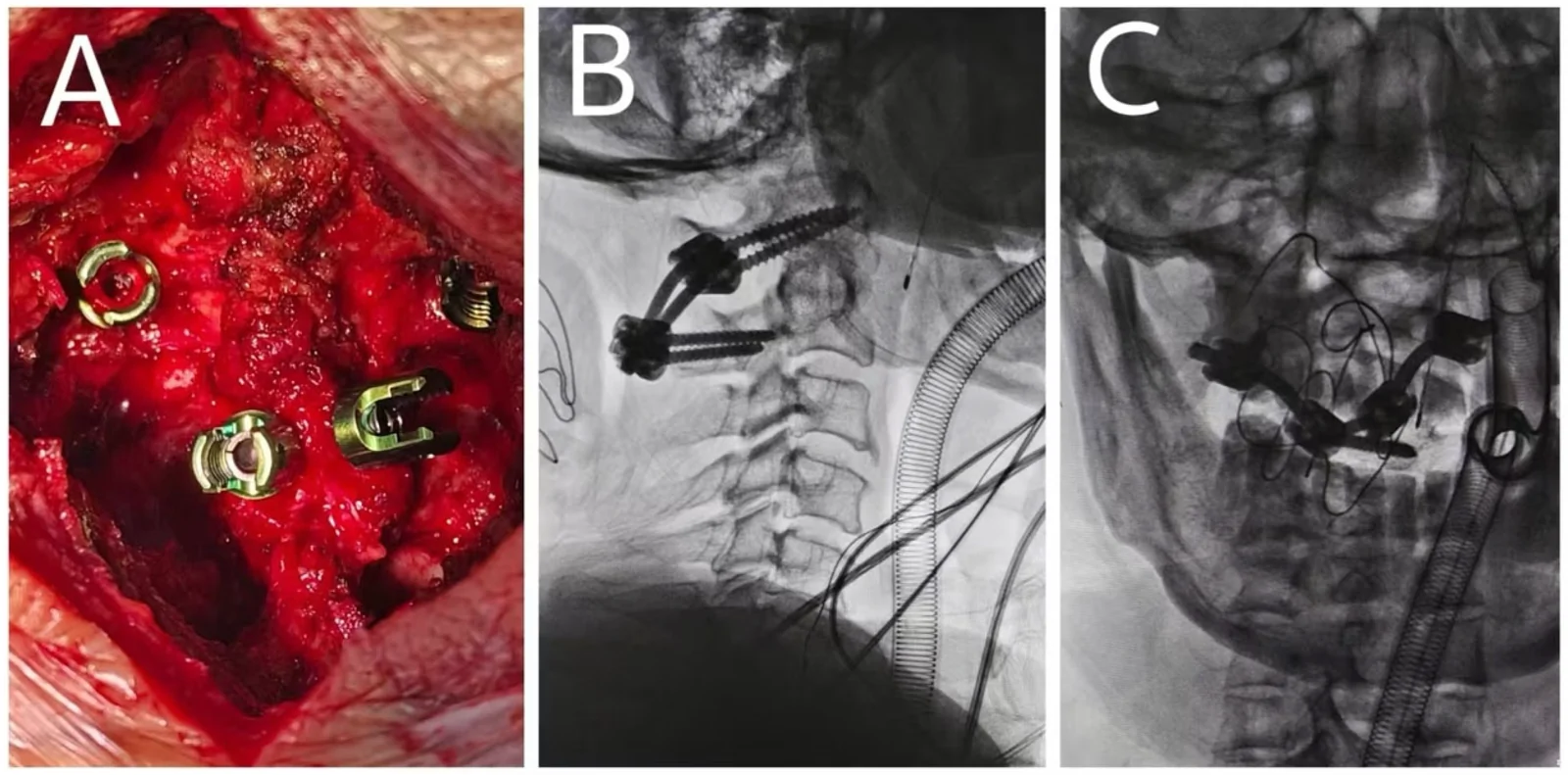
Intraoperative photograph and fluoroscopic images of C1 lateral mass screws combined with C2 laminar screw fixation: (**A**) intraoperative view showing implanted C1 lateral mass screws and C2 laminar screws; (**B**) lateral and (**C**) anteroposterior fluoroscopic radiographs of atlantoaxial screw placement.

Currently, the most widely adopted screw–rod system covers four screw trajectories: C1 lateral mass screws, C1 pedicle screws, C2 laminar screws, and C2 pedicle screws [20]. C1 pedicle screws yield the highest pullout strength with less intraoperative bleeding and neural irritation, making them the first choice for atlas fixation [7,21]. C2 pedicle screws provide twice the holding power of isthmic screws and can achieve a nearly 100% fusion rate and allow for intraoperative reduction, which renders them the primary fixation option for the axis [12,22]. C1 lateral mass screws require extensive soft tissue dissection and frequently result in greater postoperative occipital neuralgia [23,24]. Although crossed C2 laminar screws avoid injury to vertebral arteries, they carry a high risk of canal penetration in osteoporotic bone. An insufficient grafting area impairs osseous fusion; therefore, this technique is only used as an alternative [25]. A representative case of atlantoaxial stabilization using C1 lateral mass screws combined with C2 laminar screws, one of the mainstream conventional posterior atlantoaxial fixation constructs, is shown in Figure 1.

All these conventional fixation implants use standard 3.5 mm single-thread titanium screws without any bioengineering modifications to account for low bone mineral densities, resulting in prominent mechanical defects at the bone–screw interface.

### Review Framework

**Figure 2:**
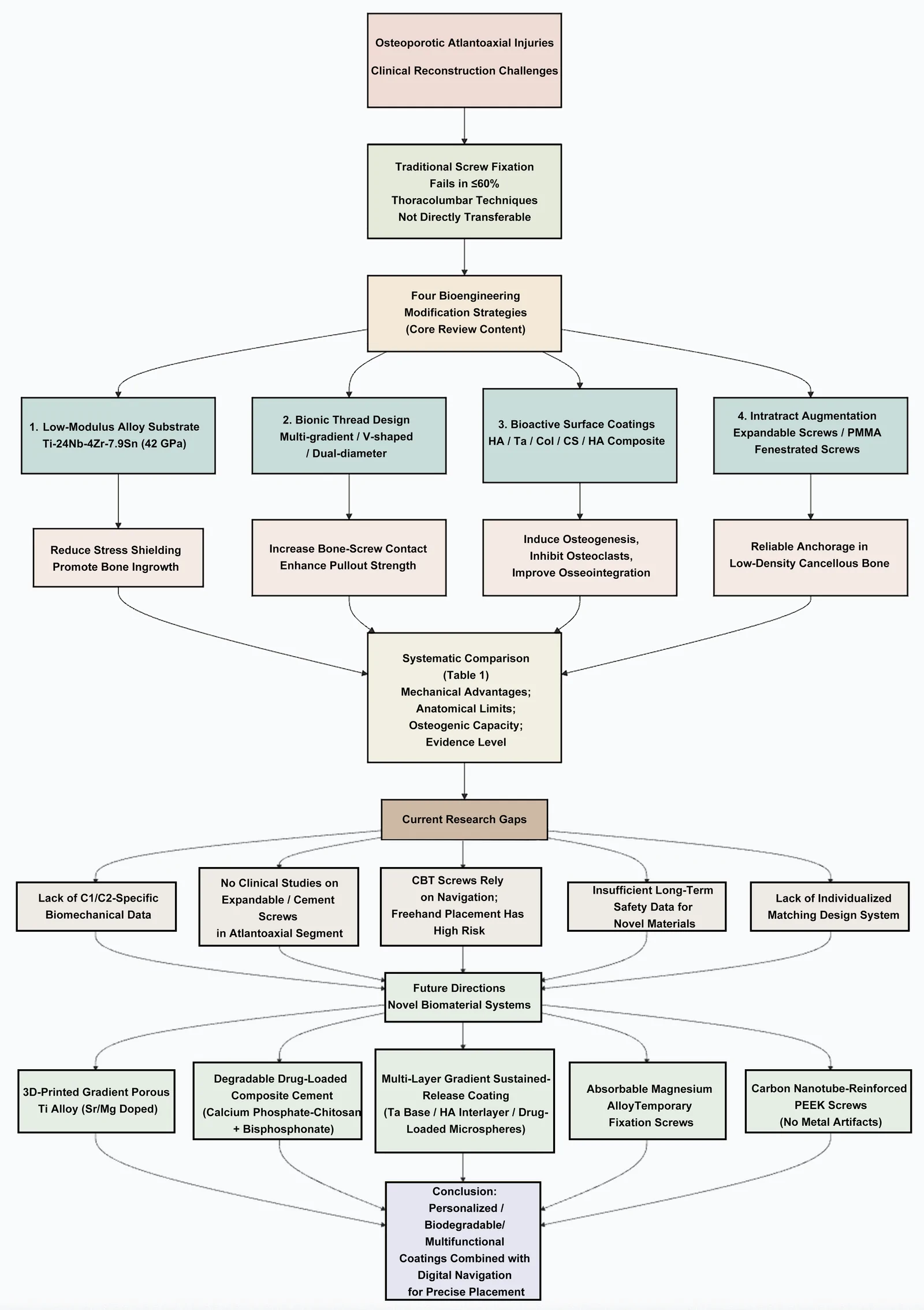
Schematic of the review’s logical framework for bioengineering optimization of atlantoaxial screws in osteoporotic patients: clinical reconstruction challenges, four major screw modification strategies, standardized multi-dimensional comparison criteria, current research gaps, five prospective novel biomaterial systems, and the concluding perspective on personalized, biodegradable, multi-functional implants combined with digital navigation (Table 1).

The overall logical framework of this review is summarized in Figure 2, which illustrates the systematic clinical challenges in osteoporotic atlantoaxial reconstruction, four core bioengineering screw modification strategies, systematic comparative evaluation indexes, unsolved research limitations, five novel biomaterial development directions, and the final integrated research conclusion.

## 3. Bioengineering Modification Technologies for Screws for Osteoporosis Patients

### 3.1. Screw Substrate Modification and Novel Low-Elastic-Modulus Alloys

Titanium alloy and stainless steel are the two predominant materials for orthopedic screws in current clinical practice. In recent years, titanium alloys have gradually replaced stainless steel as the primary implant material. This transition is supported by experimental evidence demonstrating that titanium alloys possess favorable biocompatibility and a low elastic modulus. These properties facilitate osseointegration between bone tissue and the implant, thereby improving biomechanical stability. In addition, titanium alloys exhibit excellent imaging properties in radiographic examinations [26]. Furthermore, pedicle screws made of titanium alloy can achieve comparable pullout resistance to conventional stainless-steel screws.

In a comparative material study conducted by Kalasauskas et al. [27], the low elastic modulus of titanium alloys effectively mitigated stress shielding. In addition, titanium pedicle screws achieved significantly stronger mechanical interlocking at the bone–screw interface and superior torsional strength. This advantage originates from improved bone ingrowth into the implant surface. Titanium alloys also produce minimal artifacts during magnetic resonance imaging (MRI) and computed tomography (CT), which greatly reduces imaging interference. Accordingly, titanium pedicle screws are more suitable for elderly osteoporotic patients with diminished osteogenic capacity.

Ti-6Al-4V is the most widely used material for clinical pedicle screws, with an elastic modulus reaching 110 GPa, which is far higher than that of native cortical bone. The modulus mismatch between pedicle screws and vertebral bone becomes even more pronounced in osteoporotic patients. Such patients have reduced bone stiffness and prolonged bone healing time, which requires long-term screw fixation and inevitably elevates the risk of screw loosening.

To address this challenge, Shi et al. [28] developed a novel expandable pedicle screw (L-EPS) fabricated from Ti-24Nb-4Zr-7.9Sn, a new titanium alloy with an elastic modulus of only 42 GPa. In their in vivo experiments, the implants were implanted into ovariectomized osteoporotic sheep. At 12 months after ovariectomy, the L-EPS group exhibited significantly higher axial pullout strength. Micro-CT reconstruction demonstrated greater bone formation surrounding L-EPS screws with more uniform bone distribution. Histological examinations revealed that newly formed bone grew along the expandable slots and extended toward the central region of the EPS. Moreover, the L-EPS group exhibited a larger contact area at the bone–screw interface, which was accompanied by less fibrous tissue ingrowth.

Previous studies have confirmed that reducing the modulus mismatch between titanium alloy implants and vertebral bone can optimize the stress distribution and further improve the long-term fixation stability of pedicle screws. Therefore, a promising research direction for future investigations is to develop titanium alloy screws with mechanical properties tailored to the elastic modulus of the host bone in individual patients.

### 3.2. Bionic Biomechanical Optimization of Screw Structure

#### 3.2.1. Multi-Gradient Thread Bionic Design

In terms of thread profile design for pedicle screws, most commercially available clinical screws adopt a single-thread configuration [29]. Although this design can meet conventional fixation requirements under normal bone conditions, pullout resistance tends to decline significantly when these screws are implanted into osteoporotic bone [30]. To achieve reliable anchorage in low-density bone, numerous researchers have explored double-thread, triple-thread, and multi-thread structures in recent years. The fundamental principle of these modified thread designs is to enlarge the contact area and frictional force between the screw thread and host bone in order to increase pullout strength and enhance the long-term stability of internal fixation.

Comparative experiments between conventional and double-threaded screws have demonstrated that double-threaded screws feature not only faster insertion speeds but also superior pullout resistance. This advantage helps reduce complications such as screw loosening and pullout in osteoporotic patients [31]. Furthermore, the two screw types in that study had different outer diameters; if the diameter was standardized, the difference in pullout strength would be even more remarkable.

Chen [31] performed surgical treatment using cortical bone screws (single-threaded and double-threaded designs) on 159 patients with degenerative lumbar disorders. The clinical data revealed that double-threaded screws achieved a higher fixation strength and significantly improved structural stability, accompanied by a lower incidence of screw loosening compared with the single-threaded counterparts.

Building upon these findings, Salunke et al. [32] proposed a novel triple-threaded screw with variable thread widths at the tip, shaft, and tail. Biomechanical pullout tests showed that the anchoring strength of the triple-threaded screw was approximately 10% and 5% higher than that of the single- and double-threaded screws. Under identical fixation settings, the maximum pullout force ranked from high to low was the triple-threaded screw, the double-threaded screw, and the single-threaded screw. This outcome confirms that adjusting the screw thread pitch can effectively enhance the bone-holding capacity of pedicle screws.

Cancellous bone screws are characterized by a large thread pitch, deep threads, and a relatively small core diameter. In contrast, cortical bone screws possess a small thread pitch, shallow threads, and a larger core diameter [33,34]. In spinal pedicle screw fixation, the anchorage force mainly originates from the cortical bone within the pedicle, while cancellous bone in the vertebral body only makes a limited contribution. Thus, it can be reasonably inferred that fine threads on the proximal segment inside the pedicle would improve screw anchorage, whereas conventional coarse threads at the screw tip achieve better fixation within cancellous bone.

Apart from thread profile, the geometry of the screw shaft, either cylindrical or conical, is another critical determinant of fixation strength. Lorenz et al. [35] reported that double-diameter screws exhibited comparable insertion torque and pullout strength to cement-augmented standard pedicle screws. Double-diameter screws can therefore effectively increase pullout resistance and insertion torque.

Kim et al. [36] manufactured nine types of pedicle screws with varying inner shaft profiles (cylindrical or conical), outer shaft profiles (cylindrical or conical), and thread configurations (V-shaped, sawtooth, and square) and conducted pullout tests on osteoporotic bone models. The experimental results indicated that screws with V-shaped threads achieved the highest pullout strength, and those with square threads had the lowest values. Regardless of bone mineral density, the pedicle screw with an outer cylindrical structure, an inner conical structure, and V-shaped threads yielded the maximum pullout load.

Comprehensive experimental results have demonstrated that square-thread and V-shaped-thread screws have distinct mechanical merits. Square-thread screws display superior torsional resistance, whereas V-shaped-thread screws achieve better anti-pullout performance. Optimizing the thread profile to expand the contact area with bone tissue can significantly enhance screw anchorage. In addition, rational adjustment of thread pitch can result in more favorable compression of trabecular bone, which generates greater interfacial friction and further improves fixation strength.

At present, most studies investigating the stability of screws with variable thread pitches focus on the thoracolumbar spine. Despite considerable anatomical differences between the atlas and the thoracolumbar vertebrae, both structures consist of cortical bone of varying thicknesses combined with cancellous bone. Accordingly, screws with a graded thread pitch can better match the anatomical characteristics of these vertebral segments.

#### 3.2.2. Optimization of Screw Diameter and Insertion Length

Numerous studies have confirmed that screw diameter is a major determinant of biomechanical strength. In general, larger screw diameters correspond to higher biomechanical stability [37,38]. Kueny et al. [39] conducted biomechanical tests on 39 osteoporotic bone specimens and found that 6.5 mm screws achieved a 5% improvement in fatigue strength and a 24% significant increase in pullout resistance compared with 5.5 mm screws.

Roch et al. [40] used twelve human lumbar vertebral specimens, including six with normal bone mineral densities and six with osteoporosis. A short 35 mm screw and the longest possible non-penetrating screw were randomly implanted on each side. A sinusoidal cephalocaudal cyclic load was applied at a frequency of 0.5 Hz, starting from 100 N and with an incremental load of 1 N per cycle, until screw head displacement reached 5.4 mm. In intact vertebrae, there were no significant differences in fatigue load between short screws (315.6 ± 148.7 N) and long screws (309.0 ± 138.3 N). In osteoporotic vertebrae, long screws exhibited markedly higher fatigue resistance (230.9 ± 55.0 N) than short screws (175.1 ± 45.5 N; *p* = 0.045). Since the overall fixation strength deteriorates in osteoporotic bone, longer screws can provide substantially enhanced stability.

Moreover, Chua et al. [41] demonstrated that screws inserted to 80.0% of the vertebral longitudinal axis achieved better and more reliable stability than those inserted to only a 50% depth. Increasing the insertion depth of pedicle screws can therefore improve fixation stability to a certain extent. Concurrent enlargement of screw diameter and insertion depth could lead to remarkable increases in insertion torque and pullout strength [42,43].

The allowable screw diameter is limited by the anatomy of the upper cervical spine, and 3.5 mm has become the standard diameter for clinical atlas screws. Zhang et al. [44] reported that the unique anatomical morphology of the atlas prevents the placement of 4.0 mm screws. However, Lenz et al. [45] obtained different conclusions. They retrospectively analyzed atlas CT images from 500 patients. Among these subjects, 342 patients (68.4%) had a posterior arch height greater than 4 mm, while the remaining 158 patients (31.6%) had a posterior arch height below 4 mm. Clinical trials further confirmed that C1 pedicle screws can still be safely inserted even when the pedicle height is less than 4 mm or 5 mm [46]. Consequently, preoperative CT measurement of pedicle height is essential for evaluating the feasibility of screw anchorage.

Barsa P et al. [47] performed atlas CT scans on 42 healthy volunteers to compare the implantation feasibility of 3.5 mm and 4.0 mm pedicle screws. The results showed that 86.9% of volunteers were eligible for 3.5 mm screws, whereas 59.5% could accommodate 4.0 mm screws. For patients suitable for larger implants, 4.0 mm screws are hypothesized to possess higher pullout resistance than 3.5 mm screws. Accurate preoperative morphometric measurement is therefore critical to surgical planning.

#### 3.2.3. Dual-Cortical Penetrating Screw Configuration

Osteoporotic cancellous bone suffers from poor mechanical bearing capacity, so conventional unicortical pedicle screws fail to achieve sufficient bone purchase, leading to a high risk of fixation failure. Common clinical improvement strategies include increasing the screw lateral angulation, adopting screws with larger diameters or longer lengths, and performing cement augmentation within the screw tract. Nevertheless, excessive lateral angulation carries potential risks such as screw penetration into the spinal canal and neurological injury.

Yasuyuki et al. [48] established 27 synthetic osteoporotic lumbar models (L3), into which unicortical and bicortical screws were inserted along three trajectories (straight anterior, cephalad, and caudad directions). The maximum insertion torques of unicortical screws along the three trajectories were 144.4, 143.1, and 148.9 Ncm, while those of bicortical screws reached 205.5, 156.2, and 207.8 Ncm, respectively. The bicortical screws produced significantly higher insertion torque than unicortical screws (*p* < 0.001). The pullout strengths of bicortical screws along the straight anterior, cephalad, and caudad trajectories were 703.3, 783.9, and 981.3 N, respectively. Liu [49] further illustrated the anatomical limitations of bicortical screw placement, finding that the risk of great vessel injury can only be minimized when screws are inserted with zero medial angulation at the L1–L3 segments; vascular complications remain unavoidable under other insertion angles.

Although bicortical screws exhibit excellent anchoring performance, their clinical application is restricted by anatomical constraints, which result in elevated risks of intraoperative neurovascular injury. Clinical and biomechanical studies focusing on bicortical screw fixation for the atlantoaxial segment remain scarce, and their application protocol still needs further improvement.

#### 3.2.4. Cortical Bone Trajectory (CBT) Screws

Conventional pedicle screws (PSs) represent the gold standard for spinal internal fixation. Nevertheless, bone loss in osteoporotic patients severely impairs screw anchorage, leading to a high incidence of complications, including screw loosening, pullout, and breakage, which remains a major clinical challenge [50,51,52]. At present, two primary strategies are adopted clinically to enhance fixation stability: extending the fusion segments and implementing cement augmentation of the screw tract. However, both methods have notable drawbacks. The exothermic polymerization of bone cement may cause thermal injury to the spinal cord and nerve roots. Other adverse events include material fatigue and fragmentation, cement extravasation, and vascular embolism. Moreover, extended fixation segments prolong operative time, increase intraoperative blood loss, and raise the risk of postoperative complications.

To overcome these limitations, Santoni’s group proposed the cortical bone trajectory (CBT) screw technique based on conventional pedicle screws in 2009 [53]. This trajectory runs laterally in the transverse plane and cephalad in the sagittal plane. The core design principle is to maximize the contact area between the screw and high-density cortical bone to improve the overall mechanical rigidity of the construct [54,55,56].

In recent years, the clinical application of CBT screws has been continuously expanded [57]. Multiple controlled trials have verified that the overall complication rate of CBT fixation is lower than that of conventional pedicle screw systems [58,59]. Compared with traditional screw placement, the entry point of CBT screws is located more medially, and most threads are concentrated on the proximal segment of the screw. This structural feature enlarges the contact area with cortical bone and achieves superior anchorage performance [56,60]. Benefiting from the outstanding mechanical strength of cortical bone, this technique markedly reduces the risk of screw loosening due to osteoporosis. Meanwhile, it causes less surgical trauma and shortens the operative duration, which facilitates postoperative recovery [61,62].

Nevertheless, the narrow and intricate anatomical morphology of the atlantoaxial segment presents significant technical difficulties in freehand CBT screw insertion, which greatly hinders its clinical popularization for upper cervical spine surgery [63,64]. Digital auxiliary technologies, such as spinal surgical robots, intraoperative fluoroscopic navigation, and 3D-printed guiding templates, can dramatically improve screw placement accuracy and safety and overcome the shortcomings of freehand manipulation [65,66].

At present, CBT screws are mainly applied in thoracolumbar pathologies as a minimally invasive and highly efficient fixation strategy. With the continuous upgrading of navigation systems and intelligent surgical equipment, this technique will likely enjoy broader application prospects in the treatment of atlantoaxial instability complicated by osteoporosis, offering an innovative therapeutic option for patients with low bone mass.

### 3.3. Engineering of Bioactive Composite Coatings on Screw Surfaces

Pathological studies have demonstrated that the absence of osseointegration at the bone–screw interface markedly elevates the risk of screw loosening [67,68,69]. Therefore, effectively inducing and accelerating new bone formation around the implant to enhance intravertebral fixation stability has become a major research field in spinal surgery.

In recent years, a variety of surface coating technologies have been applied to pedicle screws to strengthen the bonding between implants and host bone. Hydroxyapatite (HA) coatings are widely used due to their excellent biocompatibility and osteoinductivity. By mimicking the chemical composition of human bone, HA coatings facilitate the adhesion and proliferation of osteocytes on screw surfaces, eventually achieving solid osseointegration.

Moreover, coating materials such as titanium (Ti), tantalum (Ta), collagen/chondroitin sulfate (Col/CS), Col/CS/HA, allogeneic bone particles (ABPs), calcium phosphate cement (CPC), and demineralized bone matrix (DBM) can effectively improve the biomechanical stability of pedicle screws [70,71]. Previous studies have indicated that implanting solid granules into vertebral bodies is safer than injecting liquid materials because liquid cement extravasation outside the vertebra may lead to vascular embolism [72,73]. Accordingly, the implantation of HA particles carries a lower risk of embolism compared with injected PMMA bone cement [74]. Although intraoperative embolism remains a potential complication in spinal surgery, HA particles are regarded as a safer alternative to PMMA and can strengthen percutaneous pedicle screw fixation [75].

Kanno et al. [76], in a study involving 32 patients, found that after HA particle augmentation of percutaneous pedicle screws, the incidence of screw loosening was only 5.8%, which is lower than previously reported rates. No implant failure or revision surgery was recorded, confirming that this augmentation method improves fixation stability.

Liu et al. [77] prepared four groups of titanium alloy screws with different coatings: collagen/chitosan (Col/CS), HA, Col/CS/HA composite, and no coating (control). These implants were randomly inserted into the pedicles from L2 to L5 in sheep models and retrieved three months later. Under non-loading conditions, titanium pedicle screws with extracellular matrix coatings induced robust new bone formation and enhanced intravertebral biomechanical stability. The screws with the Col/CS/HA composite coating exhibited the strongest osteoinductive capacity and the highest pullout strength. Under cyclic loading, abundant connective tissue formed around the screws in all groups, and no significant intergroup differences were observed.

Titanium alloys have become the dominant material for pedicle screw fabrication due to their inherent low elastic modulus [78]. In contrast, tantalum (Ta) can inhibit osteoclast activity and support greater cell adhesion and proliferation compared to titanium alloy. Micro-CT, histological, and biomechanical analyses have verified that Ta-coated screws can markedly accelerate trabecular bone regeneration. These findings confirm that Ta is a potent osteogenic promoter, and Ta coatings are an effective modification strategy for titanium implants.

Jia et al. [71] evaluated the biomechanical performance of pedicle screws augmented using three materials on 120 human cadaveric vertebrae: allogeneic bone particles (ABPs), calcium phosphate cement (CPC), and demineralized bone matrix (DBM). The measured parameters included insertion torque, pullout strength, number of failure cycles, and ultimate failure load. All three materials improved screw stability, with CPC achieving the best augmentation effect, followed by ABPs, while DBM produced the weakest improvement. For revision operations, CPC remained the first choice; ABPs could be used as a substitute, whereas DBM was not recommended.

Future research can focus on optimizing combinations of different coating materials. This strategy could increase the bonding strength between screws and bone tissue while alleviating inflammatory reactions triggered by the implant. For instance, composite coatings can be fabricated by blending materials with favorable biocompatibility and osteoinductive substances to further improve screw fixation efficacy under osteoporotic conditions.

In addition, developing customized coatings tailored for specific patient populations, especially elderly osteoporotic patients, to match their unique biomechanical requirements represents another important research avenue.

### 3.4. Internally Augmented Bioengineered Pedicle Screws

#### 3.4.1. Expandable Biomimetic Screws

The pullout resistance of pedicle screws is strongly correlated with bone mineral density. Osteoporotic patients generally have low bone density, which reduces the anchoring capacity of the host bone and eventually leads to screw loosening. The unique structure of expandable screws enlarges the contact area between the implant and vertebral cancellous bone, thereby improving biomechanical stability. Several biomechanical experiments have confirmed that expandable pedicle screws achieve superior pullout stability compared with conventional pedicle screws in osteoporotic spinal bones [79,80].

Chen et al. [81] designed a 6.5 mm expandable pedicle screw with four centrosymmetric fins on its anterior segment. When the fins were fully deployed, the maximum outer diameter of the screw expanded to 9.5 mm. Biomechanical tests performed on osteoporotic composite bone models revealed that the pullout resistance of the expandable screw was comparable to that of a conventional screw of the same diameter augmented with 2 mL bone cement.

Aycan et al. [82] conducted experiments using polyurethane foam with densities of 0.16 and 0.64 g/cm^3^ and demonstrated that an expandable-shell pedicle screw exhibited equivalent pullout strength to hollow pedicle screws filled with bone cement. Moreover, the expandable screw achieved higher pullout resistance than cement-augmented screws in intact vertebral bone.

Fu et al. [83] performed a two-year follow-up study on 27 patients with osteoporotic degenerative spinal deformity, in whom a total of 376 expandable pedicle screws were implanted. No screw breakage or loosening was observed during the follow-up period. These clinical outcomes verify that expandable pedicle screws offer satisfactory therapeutic effects for this patient population. They can provide reliable fixation, improve radiographic and clinical outcomes, and maintain a low incidence of screw-related complications.

Expandable pedicle screws carry a risk of vertebral bone destruction during removal, which may lead to injury of nerve roots or the dura mater. Despite this limitation, expandable pedicle screws remain a viable alternative to standard pedicle screws and cement-augmented screws for osteoporotic spinal surgery. At present, expandable screws have been preliminarily applied clinically for osteoporotic patients and have achieved better outcomes than conventional screws. Nevertheless, given the relatively short clinical history and limited application scope, their long-term therapeutic efficacy still requires further clinical observation and verification.

#### 3.4.2. Cannulated Screws with Lateral Fenestrations for Cement Augmentation

Perfusion-type pedicle screws feature a hollow shaft with lateral fenestrations. Bone cement can be injected through these structures to significantly enhance fixation stability. Characterized by rapid setting and high mechanical strength after solidification, bone cement forms tight bonding between the screw and cancellous bone, which strengthens the interfacial integration between the implant and bone tissue. At present, this screw design has been widely adopted for the treatment of osteoporotic spinal disorders [84].

Erdem et al. [85] demonstrated that cement augmentation of pedicle screws significantly increased pullout force by 96% to 262%, while lateral bending stiffness increased by 153%.

In the study conducted by Mueller et al. [86] involving 474 PMMA-augmented pedicle screws implanted into 237 vertebral bodies in 98 patients, no symptomatic cement extravasation or pulmonary embolism was recorded. However, asymptomatic perivertebral cement leakage was observed in 165 vertebrae (34.8%) in 88 patients (89.8%), and the most common leakage site was the perivertebral venous plexus. In addition, four patients (4.08%) developed asymptomatic pulmonary embolism. The authors concluded that adjusting the size and diameter of the lateral holes in cannulated pedicle screws could reduce the risk of cement extravasation. In general, cement leakage was prevalent yet mostly subclinical.

Tan et al. [87] developed a novel cannulated pedicle screw with progressively smaller lateral hole diameters. Six side holes were distributed along the screw shaft, with diameters tapering from the tip toward the proximal end. The FPS-B design, which forms a cylindrical PMMA reinforcement around the screw, was regarded as the optimal option for osteoporotic vertebrae. It yielded a higher pullout strength than the CPS design and lowered cement leakage risk compared with the FPS-A design, which produced a conical cement distribution. The study recommended 1.5 mL PMMA as the optimal augmentation volume, though this finding still needs to be validated by numerical simulations and in vivo physiological experiments.

Despite the studies mentioned above, there are no published studies on cement-augmented screws applied to the atlantoaxial segment, and cement extravasation remains the primary safety concern. Modified designs such as cannulated pedicle screws with gradually tapering lateral holes can enhance screw–bone bonding while minimizing the risk of cement leakage. With further technological advances, optimized cement-augmented screw designs are expected to address these challenges and improve clinical outcomes.

To compare the biomechanical characteristics, osteogenic capacity, anatomical restrictions, and clinical research maturity of all mainstream screw bioengineering modification strategies for osteoporotic atlantoaxial fixation, a comprehensive comparative summary is provided in Table 1.

**Table 1:** Comparison of six typical bioengineering-modified screw systems for osteoporotic atlantoaxial internal fixation, including substrate alloys, structural design, surface coatings, and intratract augmentation techniques. The table systematically compares their mechanical superiority, unique anatomical limitations in the C1–C2 segment, osteogenic performance, and the existing research evidence level. Abbreviations: CBT, cortical bone trajectory; PMMA, polymethyl methacrylate; HA, hydroxyapatite; Col/CS, collagen/chitosan; BMD, bone mineral density; Ti, titanium; C1/C2, atlantoaxial segment.

Modification Category	Specific Optimization Scheme	Core Biomechanical Advantages	Key Limitations for Atlantoaxial (C1/C2) Application	Osteogenic Performance	Clinical Evidence Level
Low-modulus alloy substrates	Ti-24Nb-4Zr-7.9Sn low-modulus titanium alloy (42 GPa)	Reduce stress shielding; uniformly distributes stress; larger bone–screw contact area; superior torsional and pullout strength	Narrow C1 pedicle space limits screw outer diameter; insufficient long-term human in vivo safety data	Promote trabecular bone ingrowth into screw surface; less fibrous tissue encapsulation	Osteoporotic animal (sheep) model + in vitro biomechanical tests; lacks C1/C2 clinical cohort data
Bionic thread structural optimization	Multi-gradient thread (double/triple/variable pitch); V-shaped thread; dual-diameter shaft	Expands bone-thread contact area; high anti-pullout capacity; V-thread showed maximum pullout load; anchoring force: triple-thread > double-thread > single-thread	Standard C1 screw diameter fixed at 3.5 mm; limited space prevents use of large thread height; anatomical variation in atlas posterior arch restricts oversized thread design	Improves trabecular compression around threads; enhances interfacial friction without obvious osteogenic promotion	Thoracolumbar cadaver and finite element tests; few upper cervical-specific verification studies
Screw dimension and trajectory optimization	Larger diameter (4.0 mm); longer insertion depth; bicortical trajectory; CBT screws	Longer screw improves fatigue resistance under low BMD; bicortical design greatly raises insertion torque and pullout force; CBT screws maximize cortical bone contact	C1 posterior arch height varies between individuals (only 59.5% of patients can accommodate 4.0 mm screws); bicortical screw implantation risks vertebral artery/vascular injury; freehand CBT placement has high neurovascular injury risk in the narrow upper cervical corridor	No direct osteogenic effect; only strengthens mechanical interlock between screw and cortical bone	CBT screws: lumbar meta-analysis; bicortical screws: in vitro lumbar tests; very little C1/C2 clinical data
Bioactive surface coatings	HA, tantalum, Col/CS/HA composite coatings	HA induces osteocyte adhesion; Ta inhibits osteoclast activity; composite coating induces optimal osteoinductivity	Small-diameter C1/C2 screws lead to thin coatings; plasma-sprayed HA easily peels under cervical rotational cyclic load; coating technology complexity raises manufacturing costs	Composite Col/CS/HA > Ta coating > single-layer HA coating; pure Ti substrate without coating showed poorest bone integration	Animal (pigs and sheep) experiments; small-sample lumbar percutaneous screw clinical observations
Expandable biomimetic screws	Centrally split expandable pedicle screws with expansion fins	Expand outer diameter inside cancellous bone; pullout strength equivalent to cement-augmented standard screws	Difficult to adjust expansion range in tiny C1 vertebral body; high risk of vertebral bone destruction during revision removal; may compress vertebral artery	Expansion slots provide space for new bone growth along fins; uniform bone distribution around implant	2-year lumbar degenerative deformity follow-up; no published clinical atlantoaxial research
Fenestrated cannulated screws with cement augmentation	Lateral gradient-tapering hole, cannulated screws + PMMA perfusion	Pullout force increased by 96–262%; higher bending stiffness; forms solid bone–screw bonding	Severe hidden risk of PMMA cement leakage that could compress spinal cord/vertebral artery; venous cement embolism risk; no standardized injection volume for C1/C2	PMMA lacks biological activity; cannot promote bone regeneration; only mechanical reinforcement	Large lumbar patient cohort data; no atlantoaxial cement augmentation clinical trials

## 4. Current Research Gaps in Osteoporotic Atlantoaxial Implant Optimization

Most biomechanical verification data for modified screws are derived from thoracolumbar vertebral models. Few in vitro cadaveric tests, finite element analyses, and large-animal in vivo experiments have been specially designed for the C1–C2 segment’s unique anatomical structure, and there is a lack of reference data accounting for upper cervical bone morphology and risks to the surrounding neurovasculature.

Expandable pedicle screws and PMMA cement-augmented fenestrated screws have achieved satisfactory effects in thoracolumbar osteoporosis treatment, but relevant clinical trials, long-term follow-up data, and standardized safe operation protocols for atlantoaxial fixation are still lacking. Moreover, cement leakage and vertebral artery/spinal cord compression risks cannot be quantitatively evaluated.

Cortical bone trajectory (CBT) screws rely heavily on intraoperative navigation or 3D-printed guiding templates to guarantee placement accuracy. Freehand CBT screw implantation into C1/C2 has an extremely high failure risk, while cost and equipment limitations restrict its wide clinical adoption.

Novel modified materials, including low-modulus β-titanium alloys, tantalum coatings, composite extracellular matrix coatings, and degradable magnesium alloys, only have short-term animal data. Their long-term in vivo biosafety, bone remodeling regulation effects, and anti-fatigue performance in elderly osteoporotic patients remain unclear.

There is a lack of individualized design schemes for atlantoaxial screws. There is no mature classification system for selecting screw diameter, thread type, coating, and augmentation strategy based on patient-specific bone mineral density, atlas posterior arch height, and vertebral artery anatomical variation.

The development of bioactive coatings has only focused on improving osteogenic capacity. Multifunctional composite coatings carrying anti-inflammatory, anti-osteoclast, and anti-osteoporosis drugs for upper cervical implants are still in the preliminary laboratory stage.

## 5. Prospects for Novel Biomaterials for Atlantoaxial Screw Implants

Current modified screw materials still have several inherent drawbacks. Conventional titanium implants easily cause stress shielding, single-layer coatings offer a limited osteogenic capacity, PMMA bone cement lacks biological activity, and expandable screws tend to produce bone defects during removal. Based on cutting-edge advances in bioengineering, three-dimensional additive manufacturing, and tissue engineering, five novel material systems can be developed for next-generation atlantoaxial screws tailored to osteoporotic patients.

First, 3D-printed gradient porous titanium alloys doped with strontium and magnesium can be fabricated via selective laser melting. This material enables precise tuning of elastic modulus to eliminate stress shielding. Its hierarchical pore structure facilitates bone ingrowth and supports patient-specific customization.

Second, traditional PMMA can be replaced with degradable calcium phosphate–chitosan composite cement loaded with bisphosphonate anti-osteoporosis drugs. This cement degrades gradually and releases bioactive agents to improve local bone mass and will eventually be fully replaced by newly formed bone tissue.

Third, a multi-layer gradient biomimetic composite coating can be constructed, consisting of a tantalum base layer, a hydroxyapatite interlayer, and a top layer with drug-loaded microspheres. This layered structure simultaneously strengthens implant–bone integration, induces osteogenesis, and achieves sustained local release of anti-inflammatory and anti-osteoporosis drugs.

Fourth, highly bioactive magnesium alloys can be used to fabricate miniature absorbable screws for temporary fixation at atlantoaxial bone graft sites. The implants would degrade spontaneously once bony fusion is achieved, thereby avoiding secondary surgical trauma.

Fifth, a carbon nanotube-reinforced PEEK composite can serve as the screw substrate. It possesses an elastic modulus close to that of cortical bone without metallic imaging artifacts. When combined with bioactive ceramic coatings, this material is suitable for elderly patients with craniocervical disorders requiring long-term radiographic follow-up, making it a promising material for developing dedicated C1/C2 screw systems in the future.

## 6. Conclusions

In this qualitative narrative review, we systematically described four major bioengineering modification strategies for atlantoaxial screw implants targeting osteoporotic patients: low-modulus metal substrate optimization, bionic structural redesign of screws, multifunctional bioactive surface coatings, and intratract augmentation, including expandable and cement-perfused cannulated screws. The advantages of these strategies are as follows: low-elastic-modulus titanium alloys effectively reduce stress shielding; gradient variable thread, bicortical, and cortical bone trajectory designs markedly increase the screw–bone contact area and anti-pullout mechanical strength; composite coatings based on HA, tantalum, and collagen–chitosan significantly promote osseointegration; and expandable screws and fenestrated cement screws deliver reliable anchorage reinforcement in low-density cancellous bone.

However, the narrow anatomical space of the atlantoaxial segment adjacent to the vertebral artery and spinal cord greatly limits the direct application of thoracolumbar augmentation technologies, which are accompanied by hidden risks of cement leakage and neurovascular injury. As summarized in Section 5, a series of critical research gaps remain, such as insufficient C1–C2-specific biomechanical data, the lack of long-term clinical follow-up of novel augmented screws, and immature individualized implant customization systems.

Combined with additive manufacturing and tissue engineering advances, the next generation of atlantoaxial implants should focus on five research directions: 3D-printed, gradient, porous, strontium/magnesium-doped titanium alloy substrates; degradable bisphosphonate-loaded calcium phosphate composite bone cement; multi-layer gradient sustained-drug-release biomimetic coatings; absorbable magnesium miniature screws for temporary graft fixation; and carbon nanotube-reinforced PEEK screws without metal imaging artifacts. Digital auxiliary technologies, including surgical robot navigation and personalized 3D-printed guiding templates, will further expand the clinical applicability of CBT and precise modified screw placement in the upper cervical spine.

This narrative review clarifies the biomechanical mechanisms, clinical advantages, and upper cervical application limitations of all mainstream screw optimization approaches, providing the first bioengineering-oriented systematic review for osteoporotic atlantoaxial fixation and comprehensive theoretical support for future implant material development and clinical surgical decision-making.

## Data Availability

No new data were created or analyzed in this study. Data sharing is not applicable to this article.
